# Variations in morphological traits of bermudagrass and relationship with soil and climate along latitudinal gradients

**DOI:** 10.1186/s41065-018-0068-2

**Published:** 2018-09-28

**Authors:** Jingxue Zhang, Miaoli Wang, Zhipeng Guo, Yongzhuo Guan, Yuxia Guo, Xuebing Yan

**Affiliations:** 1 College of Animal and Veterinary Science, Henan Agricultural University, Zhengzhou, 450002 China; 2 College of Animal Science and Technology, Yangzhou University, Yangzhou, 225000 China

**Keywords:** *Cynodon dactylon*, Morphology, Variation, Latitude, Soil nutrient

## Abstract

**Background:**

This complex environmental heterogeneity coupled with the long-standing history offers scenario suitable for and favoring the evolution and existence of variation of morphological traits.

**Methods:**

In this study, we measured 10 morphological traits of 310 *Cynodon dactylon* individuals sampled at 16 different locations along latitudinal gradients between 22°35′N and 36°18′N to reveal phenotypic plasticity influenced by latitude. In addition, the relationships between morphological variation and soil nutrient and climate factors were analyzed.

**Results:**

Analysis of variance, divesity examination and Mantel correlation test detected a significant effect of latitude on morphological traits. Cluster analysis and principal component analysis clearly separated the selected populations into four groups according to latitude. Larger morphological sizes of *C. dactylon* appeared at the low- and high-latitude regions. Correlation analysis indicated that high morphological variations were significantly correlated with climate factors and soil nutrient.

**Conclusion:**

This study suggests morphological variation of wild bermudagrass is greatly influenced by latitude as well as soil and climate, which could be useful resources for genetic studies and evolution.

## Background

Morphological plasticity may be of critical importance for resource acquisition by plants [1]. They change their growth form to become more flexible in favorable habitat. The morphological and developmental diversity between taxa and within each taxon is vast. Phenotypic variation observed for plants is generally a response to differences in climatic conditions that reflects adaptive evolution and phenotypic plasticity [2–6]. The *Cynodon* species is enormously variable and has become truly cosmopolitan which is originated and diversified somewhere from West Pakistan to Turkey(i.e., Turkey, Iran, Afghanistan and the West part of Pakistan) and distributed in all countries and islands between about lat 45°N and lat 45°S and penetrates to approximately lat 53°N in Europe. Perennial Bermudagrass [*Cynodon dactylon* (L.) Pers.] of the tribe Chlorideae (Poaceae) is a genetically and morphologically diverse warm-season grass widely used for pasture, forage, turf grass, soil stabilization, and remediation [7, 8]. Over the past decades, several studies have examined morphology, physiology, and biochemistry in order to assess the diversity of *C. dactylon* [9–12]. There is enormous morphological variability among the nine recognized *Cynodon* species [13]. Australia is third to Africa and Asia respectively in terms of *Cynodon* species diversity and seven *Cynodon* species currently were recognized in the Australian Biological Resources Study [14, 15]. Bermudagrass is widespread in Italy under different ecological situations, and there was wide variation in wild populations concerning morphological traits of potential interest for turf use, such as leaf size and growth habit [16].

Geographical or environmental gradient exerts the strongest adaptive selection on some related variation both in genetic structure and phenotypic traits of a species. Many of these factors include temperature, precipitation, soil nutrient availability, growing season length, photoperiod and biotic agents are directly affected by geographic position or elevation, and are therefore interrelated. Such latitudinal gradients in species richness are among the most universal features of nature and have been discussed by many authors [17, 18]. Changes in latitude have a drastic effect on the physical, chemical, and biological properties of soil and hence change the quality of soil. Regeneration and growth of vegetation occurred successfully are affected by a variety of conditions of soil [19]. It is well known that N transformations are affected by a number of soil properties, including mineralogy and texture, organic matter quantity and quality, pH, moisture, and microbial population size [20], and these soil properties can vary considerably from one turfgrass system to another [21, 22]. Soil organic matter is an important component of soil, and microbial biomass is the living and active part of the soil organic matter, the source and sink of available nutrients, builds up with increased accumulation of organic matter during soil development [23–26] . Some types of reactive N (e.g., NH_4_^+^ and NO_3_^−^) are essential to plant growth and ecosystem productivity. Due to the direct relationship between soil nutrients and quality, it is closely linked to the primary productivity of the ecosystem [27], and responsible for the nutrient cycling and development and function of the soil system [28, 29]. Latitudinally-defined climate conditions may provide specific *Cynodon* types and soil environments. The optimal resolution at which soil and *Cynodon* variables should be sensed and treated is not well defined. Scientific information on the variability and distribution of soil properties is critical for understanding ecosystem processes and making sustain-able soil, crop, and environmental management decisions [30, 31]. Strategic sampling of *Cynodon* at simply different latitude has not been conducted in China and the collection is expected to include valuable germplasm for *Cynodon* improvement and research. The objectives of this study were: (1) to examine morphological variability of *Cynodon* along latitude gradient; (2) to determine if and how differences in morphological traits of *Cynodon* were correlated with climate factors and soil nutrient. This information will facilitate the future use of germplasm in *Cynodon* breeding, genomics, conservation, taxonomy, and phylogeographical research and evaluate accurately whether natural selection and migration may allow evolutionary responses for populations to sufficiently match their new climates.

## Methods

### Plant materials and morphological evaluation

A total of 310 individual plants of *C. dactylon,* comprising of root and stem with a spacing of at least 50 m apart among each individual, were separately collected from 16 sites at different latitudes between 22°35′N and 36°18′N with similar longitude in China (Table 1). The morphological traits of each bermudagrass plants were examined on the collection site, including leaf length, leaf width, internode diameter, internode length, turf height, and reproductive branch height during August 2015. The longest leaf at the third node below the apical meristem of the stolon and erect shoot was used for the measurement of leaf length (mm) and leaf width (mm) with vernier calipers. Additionally, random stolons and erect shoots on each site were measured for internode length (mm) and diameter (mm) between the third and fourth fully extended nodes from the apical meristem. Turf height (mm) and reproductive branch height (mm) were determined quantitatively on each site. Total annual precipitation, mean annual temperature, annual maximum and minimum temperature of each collection location were provided by the China Meteorological Administration (Table 1).

**Table 1:** *C. dactylon* populations collected from different latitudes in China

Population code	Localities	Altitude/m	Latitude	Longitude	Annual average temperature/°C	Annual maximum temperature/°C	Annual minimum temperature/°C	Annual average precipitation/mm
1	Cixian	130	36°18′40″	114°11′51″	13.4	19.5	7.9	509.2
2	Huixian	120	35°29′26″	113°48′23″	14.6	20.3	9.8	586.9
3	Zhengzhou	90	34°54′04″	113°38′20″	14.7	20.3	9.9	640.8
4	Xuchang	90	34°00′30″	113°45′23″	14.6	20.3	9.9	733.5
5	Zhumadian	50	33°09′47″	114°03′45″	15.2	20.4	10.7	990.4
6	Xinyang	100	32°08′38″	113°59′46″	15.5	20.4	11.7	1106.1
7	Xiaochang	50	31°18′59″	114°02′15″	16.8	21.3	13.4	1138.0
8	Xiantao	30	30°25′48″	113°26′05″	17.0	21.2	13.8	1238.6
9	Linxiang	60	29°28′32″	113°26′48″	16.8	21.5	13.4	1582.5
10	Liuyang	90	28°09′14″	113°33′42″	17.5	22.5	13.8	1551.3
11	Youxian	90	27°00′59″	113°23′07″	18.1	22.6	14.8	1518.4
12	Guidong	810	26°03′49″	113°56′34″	15.8	21.6	12.1	1742.4
13	Renhua	90	25°05′29″	113°43′17″	19.9	25.1	16.5	1660.9
14	Yingde	50	24°10′31″	113°22′08″	21.2	25.8	18.1	1835.9
15	Guangzhou	10	22°51′48″	113°22′22″	22.8	27.2	19.6	1906.8
16	Zhongshan	0	22°35′40″	113°23′17″	22.0	25.9	19.1	1846.8

### Soil nutrients evaluation

Soil samples (0–20 cm depth) were collected separately under the canopy of the different plant of *C. dactylon* in twenty random quadrats (10 m × 10 m) at each site in August 2015. In each quadrat, the soil was randomly collected with five replicates. After carefully removing the surface organic materials and fine roots, the soil sample was air-dried at 25 °C room temperature for the estimation of soil physicochemical properties, exclusive of plant residues and ground to pass through 2 mm nylon sieves for analyzing Available Nitrogen(AN), Soil Organic Matter (SOM), Total Nitrogen (TN), pH, exchangeable Ca, Na, Mg and Available Kalium(AK). Chemical analyses were conducted following the methodologies [32]. The AN, SOM, TN and AK were measured using the Kjeldahl method, the potassium dichromate wet combustion procedure (Agricultural Chemistry Committee of China, 2006), Kjeldahl method (Agricultural Chemistry Committee of China, 1987) and flame emission spectrometry (Agricultural Chemistry Committee of China, 2004), respectively. Soil pH was measured with a glass electrode, samples having been diluted with water (the ratio of soil to water was 1:2.5). Exchangeable Ca, Mg and Na concentrations were determined on continuum-source atomic-absorption spectrometry (SpectrAA20 Varian) with soil extracts.

### Statistical analysis

One-way analysis of variance (ANOVA) procedures were used to test significant differences in soil properties, morphological characters of *C. dactylon* among latitudinal gradients. We also used morphological data to calculate Shannon-Wiener index by using software package Popgen 32 to evaluate the variation frequency and degree of variation of morphological traits of different geographical groups. The geographical distance matrix was calculated by using the arc distance between each pair of sites based on the latitude and longitude of locations. Mantel correlation coefficients between morphological traits and geographical distance matrices were calculated by using NTSYSpc version 2.10e. For each morphological variable the observe values in different latitude were subjected to a Principal Component Analysis (PCA) and Cluster Analysis (CA) using Ward’s method. Pearson’s correlation coefficient was used to check the relationships between morphological traits and environmental characters. Statistical analysis was performed using the software package SPSS13.0 for Windows (SPSS Inc. Chicago, USA). Figures were generated by Sigmaplot 10.0 (Systat Software Inc.) and the R package.

## Results

### ANOVA analysis

Descriptive statistics including mean, maximum, minimum, standard deviation and coefficient of variation (CV) for *C. dactylon* morphological data were given in Table 2. Analysis of data showed high morphological variability at different latitude. With regard to morphological traits, the CV values of leaf length, internodes length, turf height and reproductive branch height were relatively high. The CV analysis indicated that leaf length of stolon was the most variable morphological trait with a CV of 44.88% (Table 2). The leaf width and diameter were not significantly differentiated, with a CV equal approximately to 10%. Also, *Cynodon* morphological diversity has been partly determined by calculating different morphological traits Shannon-Wiener index. During *Cynodon* morphological traits, range of Shannon-Wiener index within latitude was 1.53–1.87, while range of Shannon-Wiener index among latitude was 1.91–2.16 (Table 3). High values of Shannon-Wiener index of different morphological traits showed high morphological traits diversity. Different Shannon-Wiener index along latitude gradient indicates different degree of morphological diversity. Morphological traits were significantly different along the latitudinal gradient by ANOVA analysis, suggesting a notable effect of latitude on the morphological traits (Table 4). Significant variation was also found among different soil properties (Table 5), SOM showed the greatest variability with a CV of 79.59%. In contrast, pH exhibited the lowest variability with a CV of 7.24%.

**Table 2:** Morphological features of the bermudagrass ecotypes used in the diversity analysis

	Parameter	Minimum(mm)	Maximum(mm)	Mean ± SD	CV(%)
Erect shoot	Leaf length	23.72	69.82	41.34 ± 12.91	31.24
	Leaf width	2.08	2.77	2.40 ± 0.21	8.80
	Internode length	11.24	36.04	20.49 ± 7.13	34.80
	diameter	0.73	1.04	0.86 ± 0.09	10.73
Stolon	Leaf length	18.99	84.92	39.32 ± 17.65	44.88
	Leaf width	2.24	3.03	2.55 ± 0.25	9.68
	Internode length	22.24	55.39	38.97 ± 10.04	25.76
	diameter	0.91	1.18	1.02 ± 0.09	8.45
	Turf height	76.30	269.40	129.80 ± 47.70	36.74
	Reproductive branch height	69.00	240.00	142.50 ± 48.80	34.21

**Table 3:** The Compare of diversity index in Morphological characters

Population code	Erect shoot				Stolon				Turf height	Reproductive branch height
	Leaf length	Leaf width	Internode length	Diameter	Leaf length	Leaf width	Internode length	Diameter		
1	1.2799	1.2206	1.6434	1.8344	1.5048	1.6094	1.2799	1.6957	1.5048	1.6957
2	1.7127	1.8082	1.8217	2.0331	1.8514	1.7820	1.6385	2.0558	1.5833	1.8082
3	1.4956	1.6908	1.5275	1.7737	1.6788	1.3728	1.7651	1.9434	1.4708	0.9819
4	1.6230	1.8605	1.6123	1.7771	1.6103	1.6230	1.7651	1.8174	0.9143	1.7219
5	1.8605	1.8344	1.8479	1.7036	1.8047	1.9604	1.6957	1.7510	2.0162	1.6796
6	1.8479	2.0558	1.8514	2.0162	1.5842	2.0685	1.8775	1.9434	1.6003	2.0558
7	1.2773	1.9298	1.3274	1.8775	1.2488	1.5833	1.6782	2.0685	1.4956	1.5013
8	1.8217	1.7524	1.5954	1.9831	0.9433	1.5430	1.4406	1.8945	1.7389	1.6696
9	1.2206	1.5218	1.3308	1.6788	1.5430	1.7524	1.3285	1.6365	1.1611	1.2376
10	1.8082	1.8945	1.5430	1.6788	1.4999	1.8741	1.9002	1.9172	1.6173	1.8183
11	1.5099	1.8183	1.8444	1.8479	1.3047	1.9560	1.5410	2.0558	1.3308	1.6400
12	1.8726	1.8082	1.9172	2.0685	1.7340	1.9865	1.9695	1.5353	1.8344	1.9865
13	1.7524	1.6046	1.7820	1.9992	1.4878	1.9900	1.8741	2.0855	1.7693	1.6526
14	1.9434	1.8217	1.8309	1.7912	1.5911	1.8444	1.7058	1.6604	1.8465	1.8775
15	1.9434	1.7036	1.9434	1.9158	1.5260	1.9865	1.9730	1.3728	1.4878	1.7524
16	1.5833	1.5911	1.8867	1.9434	1.5537	1.7340	2.0558	2.0127	1.5911	1.4703
Mean	1.6595	1.7448	1.7066	1.8701	1.5292	1.7916	1.7180	1.8404	1.5602	1.6593
Total	2.0338	2.0922	2.0668	2.1302	1.9116	2.1626	2.0713	2.0832	2.0474	2.0650

**Table 4:** Morphological traits of the bermudagrass for each site along a latitudinal gradient used in analysis of variance (ANOVA)

Traits	Source of variation	Sum of squares	df	F	*P*-Value
Leaf length of erect shoots	Among sites	49,676.968	15	7.669	0.000
	Within sites	126,959.6	294		
	Total	176,636.568	309		
Leaf width of erect shoots	Among sites	13.256	15	4.098	0.000
	Within sites	63.401	294		
	Total	76.657	309		
Internode length of erect shoots	Among sites	15,168.084	15	8.711	0.000
	Within sites	34,129.559	294		
	Total	49,297.644	309		
Diameter of erect shoots	Among sites	2.523	15	4.556	0.000
	Within sites	10.852	294		
	Total	13.375	309		
Leaf length of stolon	Among sites	92,917.8	15	9.562	0.000
	Within sites	190,451.495	294		
	Total	283,369.295	309		
Leaf width of stolon	Among sites	18.252	15	5.462	0.000
	Within sites	65.496	294		
	Total	83.748	309		
Internode length of stolon	Among sites	30,016.446	15	4.8	0.000
	Within sites	122,555.695	294		
	Total	152,572.141	309		
Diameter of stolon	Among sites	2.209	15	2.795	0.000
	Within sites	15.491	294		
	Total	17.7	309		
Turf height	Among sites	6810.65	15	14.538	0.000
	Within sites	9182.288	294		
	Total	15,992.938	309		
Reproductive branch height	Among sites	6688.848	15	10.975	0.000
	Within sites	11,945.886	294		
	Total	18,634.735	309		

Significant at 1% level *P* < 0.01

**Table 5:** Soil features of different latitudes used in the diversity analysis

Parameter	N	Minimum(g/kg)	Maximum(g/kg)	Mean ± SD	CV(%)
Total Nitrogen	16	0.45	1.88	1.14 ± 0.37	32.27%
Available Kalium	16	0.11	0.35	0.20 ± 0.08	41.41%
Available Nitrogen	16	0.01	0.16	0.06 ± 0.03	53.86%
Soil Organic Matter	16	11.55	105.50	28.24 ± 22.48	79.59%
Exchangeable Ca	16	1.91	8.45	3.80 ± 1.91	50.29%
Exchangeable Mg	16	0.08	0.38	0.21 ± 0.11	50.92%
Exchangeable Na	16	0.06	0.33	0.14 ± 0.07	50.96%
pH	16	5.72	7.16	6.58 ± 0.48	7.24%

### Cluster analysis

**Fig. 1:**
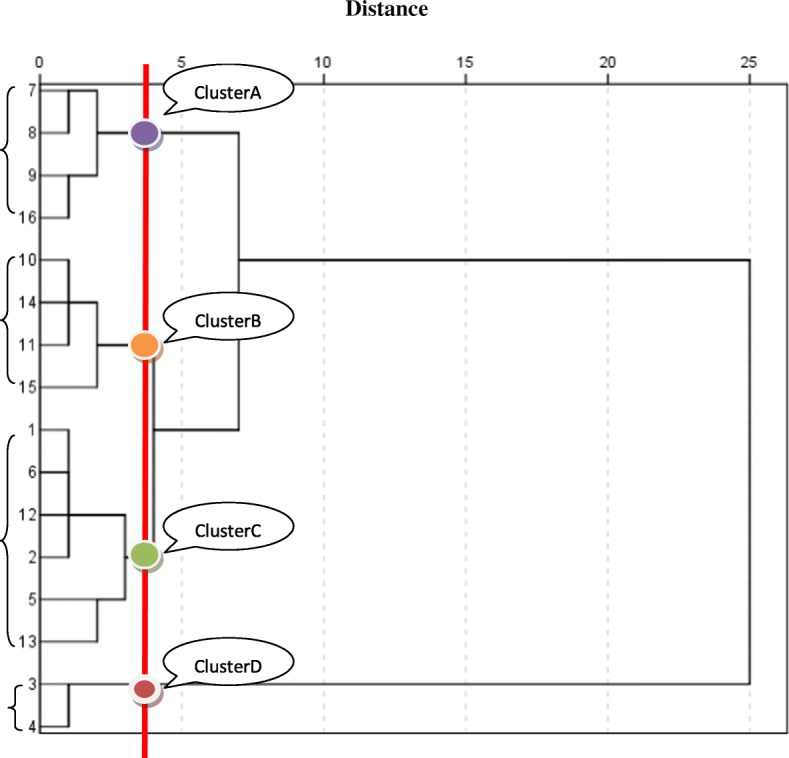
Dendrogram generated by Ward’s method of cluster analysis showing four major clusters on the basis of morphological characters of *C. dactylon* populations in China. The different colors correspond to four clusters (purple, orange, green and red circles correspond cluster A, B, C and D respectively)

PCA allowed us to find out which variables contribute most to the differences among groups (Table 6). The first two principal components (PCs) accounted for 67.47% of total variance. Dendrograms was used to graphically represent a hierarchical cluster analysis of the relationship between 16 populations based on bermudagrass morphological traits at different latitudes, respectively. From the cluster analyses, obvious effect of latitude on the morphological traits of common bermudagrass was observed. In dendrogram generated based on bermudagrass morphological traits, the 16 populations were separated into 4 clusters on the unweighted pair group mean average (UPGMA) tree (Fig. 1). This dendrogram showed that cluster A corresponded with middle latitude, cluster B corresponded with low latitude, cluster D corresponded with high latitude, while cluster C was the admixture group with low and high latitude. We attempted to estimate whether morphological traits and soil properties varied with latitudinal gradient. Based on the results, the distribution of morphological traits and soil properties within the 16 populations among the different latitudes was presented in Table 7. These four clusters were different mainly in morphological traits, with lower values in cluster A than in clusters B, C and D.

**Table 6:** Loadings of the significant morphological variables on two first principal components from analysis of *C. dactylon* morphological data

	Variable	Principal component	
		1	2
Erect shoot	Leaf length	0.156	−0.218
	Leaf width	0.157	0.195
	Internode length	0.172	−0.096
	diameter	0.114	0.302
Stolon	Leaf length	0.126	−0.257
	Leaf width	0.158	0.083
	Internode length	0.135	0.195
	Diameter	0.124	0.315
	Turf height	0.148	−0.186
	Reproductive branch height	0.171	−0.192

**Table 7:** Distribution of morphological traits of bermudagrass (*C. dactylon*) and soil characters from different latitudes

	Parameter	1	2	3	4	5	6	7	8	9	10	11	12	13	14	15	16
Erect shoot	Leaf length(mm)	47.15 ± 7.13	37.68 ± 3.99	67.51 ± 4.88	69.82 ± 9.95	40.30 ± 5.16	42.99 ± 5.45	25.98 ± 2.27	32.93 ± 4.05	23.72 ± 1.42	36.21 ± 4.11	42.23 ± 2.59	37.30 ± 4.24	33.49 ± 3.57	42.48 ± 4.25	51.53 ± 5.02	30.04 ± 2.53
	Leaf width(mm)	2.28 ± 0.25	2.53 ± 0.12	2.77 ± 0.12	2.75 ± 0.13	2.46 ± 0.10	2.33 ± 0.10	2.25 ± 0.06	2.25 ± 0.05	2.08 ± 0.07	2.29 ± 0.08	2.29 ± 0.10	2.31 ± 0.12	2.67 ± 0.11	2.43 ± 0.12	2.61 ± 0.10	2.13 ± 0.09
	Internode length(mm)	23.45 ± 2.82	25.66 ± 3.44	36.04 ± 4.17	35.67 ± 3.22	20.49 ± 1.94	20.94 ± 3.04	13.69 ± 1.60	14.46 ± 1.31	11.24 ± 0.81	16.46 ± 1.44	17.10 ± 1.76	20.42 ± 2.04	16.18 ± 1.89	16.88 ± 1.92	23.18 ± 3.28	16.00 ± 1.75
	diameter(mm)	0.79 ± 0.05	0.90 ± 0.03	0.95 ± 0.06	1.00 ± 0.06	1.04 ± 0.05	0.87 ± 0.04	0.91 ± 0.03	0.90 ± 0.04	0.76 ± 0.03	0.73 ± 0.03	0.77 ± 0.02	0.86 ± 0.06	0.94 ± 0.04	0.76 ± 0.05	0.80 ± 0.04	0.81 ± 0.04
Stolon	Leaf length(mm)	32.29 ± 6.21	30.77 ± 4.90	74.63 ± 9.33	84.92 ± 11.66	45.16 ± 8.38	35.47 ± 3.63	18.99 ± 1.74	22.88 ± 1.39	28.35 ± 2.83	32.65 ± 3.29	36.11 ± 3.17	40.18 ± 8.12	28.71 ± 3.12	39.50 ± 3.38	49.30 ± 5.43	29.21 ± 3.34
	Leaf width(mm)	2.42 ± 0.23	2.42 ± 0.11	3.03 ± 0.11	2.99 ± 0.12	2.65 ± 0.14	2.57 ± 0.13	2.25 ± 0.06	2.24 ± 0.05	2.39 ± 0.05	2.48 ± 0.08	2.53 ± 0.08	2.33 ± 0.10	2.78 ± 0.11	2.73 ± 0.12	2.70 ± 0.14	2.28 ± 0.08
	Internode length(mm)	34.47 ± 7.53	48.20 ± 4.96	51.17 ± 4.36	53.76 ± 5.16	46.42 ± 4.74	33.91 ± 3.78	29.63 ± 3.73	22.24 ± 1.84	24.49 ± 3.81	33.51 ± 3.19	55.39 ± 6.25	42.76 ± 5.04	31.50 ± 4.33	38.89 ± 5.04	36.31 ± 5.55	40.89 ± 4.55
	diameter(mm)	1.00 ± 0.08	1.03 ± 0.03	1.16 ± 0.06	1.13 ± 0.06	1.18 ± 0.05	1.06 ± 0.05	1.02 ± 0.04	1.08 ± 0.04	0.91 ± 0.04	0.93 ± 0.03	0.98 ± 0.05	1.07 ± 0.06	1.01 ± 0.04	0.92 ± 0.07	0.91 ± 0.07	0.97 ± 0.05
	Turf height(cm)	14.10 ± 1.37	14.05 ± 1.40	19.48 ± 1.47	26.94 ± 1.81	15.18 ± 2.07	13.68 ± 1.44	7.63 ± 1.05	9.98 ± 1.14	8.35 ± 0.54	10.28 ± 0.90	10.40 ± 0.70	12.50 ± 1.07	10.53 ± 1.03	13.35 ± 1.47	12.35 ± 0.94	8.90 ± 0.83
	Reproductive branch height(cm)	14.98 ± 1.74	18.47 ± 1.61	23.90 ± 0.79	24.00 ± 2.45	14.66 ± 1.30	14.48 ± 2.22	10.02 ± 0.77	11.14 ± 0.96	6.90 ± 0.48	11.14 ± 1.34	10.97 ± 0.94	13.62 ± 1.38	11.69 ± 1.24	13.74 ± 1.25	18.66 ± 1.85	9.66 ± 0.79
	Total Nitrogen (g/kg)	1.55 ± 0.04	1.14 ± 0.01	1.11 ± 0.04	1.28 ± 0.01	1.39 ± 0.04	1.20 ± 0.04	0.69 ± 0.01	1.07 ± 0.01	1.88 ± 0.00	1.05 ± 0.01	0.79 ± 0.00	0.45 ± 0.01	1.24 ± 0.04	1.28 ± 0.01	1.48 ± 0.04	0.63 ± 0.01
	Available Kalium (g/kg)	0.14 ± 0.01	0.34 ± 0.05	0.33 ± 0.01	0.24 ± 0.00	0.24 ± 0.01	0.20 ± 0.02	0.11 ± 0.00	0.13 ± 0.00	0.13 ± 0.01	0.14 ± 0.01	0.13 ± 0.01	0.17 ± 0.01	0.16 ± 0.02	0.13 ± 0.01	0.35 ± 0.03	0.21 ± 0.01
	Available Nitrogen (g/kg)	0.06 ± 0.00	0.07 ± 0.00	0.09 ± 0.00	0.07 ± 0.00	0.07 ± 0.00	0.06 ± 0.00	0.03 ± 0.00	0.06 ± 0.00	0.04 ± 0.00	0.04 ± 0.00	0.05 ± 0.00	0.01 ± 0.00	0.05 ± 0.00	0.07 ± 0.00	0.16 ± 0.01	0.05 ± 0.00
	Soil Organic Matter (g/kg)	105.5 ± 3.54	34.00 ± 0.71	17.00 ± 0.57	26.60 ± 0.00	25.10 ± 0.57	21.65 ± 0.35	11.55 ± 0.21	15.6 ± 0.42	41.95 ± 0.07	19.40 ± 0.28	15.95 ± 0.35	11.80 ± 0.28	38.5 ± 0.71	24.55 ± 0.21	26.65 ± 0.35	16.1 ± 0.57
	Exchangeable Ca (g/kg)	7.47 ± 0.07	8.45 ± 0.49	4.46 ± 0.10	4.70 ± 0.00	3.33 ± 0.11	4.63 ± 0.02	2.07 ± 0.04	3.81 ± 0.02	2.85 ± 0.04	2.15 ± 0.42	2.30 ± 0.01	1.97 ± 0.01	2.99 ± 0.01	4.65 ± 0.14	1.91 ± 0.01	3.14 ± 0.35
	Exchangeable Mg (g/kg)	0.24 ± 0.00	0.26 ± 0.00	0.36 ± 0.02	0.33 ± 0.00	0.30 ± 0.01	0.38 ± 0.00	0.35 ± 0.01	0.18 ± 0.00	0.19 ± 0.01	0.12 ± 0.03	0.12 ± 0.00	0.08 ± 0.00	0.11 ± 0.00	0.08 ± 0.01	0.15 ± 0.00	0.12 ± 0.01
	Exchangeable Na (g/kg)	0.33 ± 0.01	0.22 ± 0.04	0.20 ± 0.01	0.15 ± 0.00	0.12 ± 0.01	0.12 ± 0.00	0.14 ± 0.01	0.09 ± 0.00	0.12 ± 0.01	0.10 ± 0.01	0.09 ± 0.00	0.08 ± 0.02	0.06 ± 0.00	0.1 ± 0.01	0.11 ± 0.00	0.25 ± 0.01
	pH	6.07 ± 0.06	6.87 ± 0.01	6.75 ± 0.01	7.08 ± 0.06	5.72 ± 0.00	7.16 ± 0.03	6.81 ± 0.01	6.14 ± 0.03	6.32 ± 0.01	5.94 ± 0.01	6.86 ± 0.03	7.02 ± 0.04	6.83 ± 0.03	7.12 ± 0.03	5.99 ± 0.04	6.56 ± 0.01

### Correlation analysis

The results of the Mantel tests showed that morphological distance matrices were not significantly correlated with geographical distance matrix (*r* = 0.0370, *P* = 0.6327). Most of the measured morphological traits at different latitude showed some kind of correlations with climate factors (Table 8). Annual average precipitation negatively correlated (*P* < 0.01) with leaf length, diameter, turf height, reproductive branch height, internodes length of erect shoots and leaf width of stolon. As for annual average temperature, it significantly correlated with diameter, turf height, leaf length of stolon, and leaf width of stolon (P < 0.01) and leaf length of erect shoots (*P* < 0.05). Beyond that, annual minimum temperature negatively correlated with reproductive branch height and internodes length of erect shoots (P < 0.05). There were positive relationships between climate factors and Diversity index. The similar distribution patterns of morphological traits and soil properties indicated their high correlation. Correlation coefficients between morphological traits and soil properties are presented in Table 9. In general, contents of AK, AN and soil exchangeable Mg had significant effects on the bermudagrass morphological traits. The soil available kalium content was dramatically correlated with internodes length of erect shoots (*r* = 0.708), leaf width of erect shoots (*r* = 0.659) and reproductive branch height (*r* = 0.763); and positively with leaf length of stolon (*r* = 0.559) and erect shoots (*r* = 0.567). AN was positively correlated with leaf length of erect shoots (*r* = 0.544), leaf width of erect shoots (*r* = 0.550) and reproductive branch height (*r* = 0.587). Meanwhile, exchangeable Mg was positively correlated with distance between internodes of erect shoots (*r* = 0.511), diameter of erect shoots (*r* = 0.601) and stolon (*r* = 0.622). Results indicate that bermudagrass morphological traits had a tendency to improve with the increase of soil exchangeable Mg, AN and available potassium content.

**Table 8:** Correlation coefficients between morphological traits of bermudagrass (*C. dactylon*) and meteorological characters

Traits	Erect shoot				Stolon				Turf height	Reproductive branch height	Diversity index
	Leaf length	Leaf width	Internode length	Diameter	Leaf length	Leaf width	Internode length	Diameter			
Annual average temperature	−.120*	−0.053	−0.089	−.181**	−.193**	−.160**	−0.002	−.181**	−.199**	− 0.061	0.253
Annual maximum temperature	−0.072	− 0.012	− 0.039	−.152**	−.140*	−.114*	0.034	−.160**	−.142*	− 0.004	0.296
Annual minimum temperature	−.159**	−0.081	−.144*	−.215**	−.235**	−.188**	− 0.039	−.210**	−.251**	−.114*	0.252
Annual average precipitation	−.221**	−0.108	−.235**	−.270**	−.298**	−.186**	−0.063	−.237**	−.344**	−.210**	0.308

*, **: significant at probability of 0.05 and 0.01, respectively

**Table 9:** Correlation coefficients between morphological traits of bermudagrass (*C. dactylon*) and soil characters

Traits	Erect shoot				Stolon				Turf height	Reproductive branch height
	Leaf length	Leaf width	Internode length	Diameter	Leaf length	Leaf width	Internode length	Diameter		
Total Nitrogen	0.158	0.162	0.084	−0.048	0.147	0.336	−0.262	− 0.169	0.194	0.130
Available Kalium	0.567*	0.659**	0.708**	0.371	0.559*	0.476	0.497	0.299	0.497	0.763**
Available Nitrogen	0.544*	0.550*	0.456	0.084	0.437	0.496	0.170	−0.037	0.338	0.587*
Soil Organic Matter	0.078	−0.050	0.090	−0.180	−0.097	− 0.017	−0.174	− 0.163	0.085	0.040
Exchangeable Ca	0.277	0.214	0.440	0.148	0.097	0.096	0.199	0.205	0.399	0.424
Exchangeable Mg	0.385	0.287	0.511*	0.601*	0.351	0.257	0.153	0.622*	0.476	0.477
Exchangeable Na	0.207	−0.071	0.357	−0.037	0.073	− 0.099	0.169	0.070	0.198	0.253
PH	0.170	0.214	0.215	0.097	0.185	0.214	0.326	0.093	0.247	0.194

*, **: significant at probability of 0.05 and 0.01, respectively

## Discussion

### Relationship between morphological diversity and latitude

The measurements indicate a considerable morphological variation in bermudagrass populations along a latitudinal gradient. The morphological variability that exists within *Cynodon* spp. is well documented [13, 33]. The relationships between latitude and phenotypic variation (particularly phenology traits such as bud set) have been established previously in other species [34–38] and first published in *Cynodon* species. The presence of high morphological variation within regions and altitudes particularly above 2000 m a.s.l. indicated the potential of each region and high altitude zones for barley improvement and conservation in the country [39]. Our study has shown that morphological traits of *Cynodon* in the high- and low-latitude populations tended to have greater size than those from the mid-latitudes in China. *Cynodon* morphological variation along environmental gradients demonstrated that their geographic patterns were shaped by environmental factors (climatic and edaphic gradients) and phylogenetic differences. Such weather condition contributed positively to rice yield by increasing the number of panicles per hill and the number of spikelets per panicle significantly [40]. Through regulating the metabolic activity and carbon allocation of plants [41], climate directly influences the morphology of leaves. In addition, climate may influence the geographic distribution of leaf traits indirectly by shaping the biogeography of the vegetation as well as soil nutrient availability [42, 43]. The larger variance occurring within sites may be the result of micro-site variability, phylogenetic or historical effects, or biotic interactions and competition [44]. Polyploidization might be a driving force behind the divergence of Chinese *Cynodon* accessions and their biodiversity [45]. Adaptation in any species requires phenotypic variation in traits, but would not occur without genotypic variation. Within a species, this adaptation can theoretically be maintained through variable selection pressures from heterogeneous environments on a number of genes [46–49]. Genetic variation was expected based on previous reports of variation among *C. dactylon* accessions from geographic areas other than China [11, 33, 50–52].

### Effect of soil nutrients on wild bermudagrass morphological diversity

Soil nutrients are important factors in evaluating soil quality, this approach, however, requires an understanding of the spatial variations of soil properties within fields. TN, available nitrogen and exchangeable Ca, Mg varied significantly among different latitudes and some soil nutrients were significantly correlated with *Cynodon* morphological characters, indicating that the phenotypic diversity presented in this species is possibly due to the variation in soil characters. Plants at high latitude need more nutrients to achieve fast growth as a response to selection imposed by lower temperatures and compressed growing seasons [53, 54]. In cold environments of high latitudes, sites can limit mineralization of organic matter, nutrient release from the mineral soil and biological nitrogen fixation. So more efficient nutrient resorption can be expected and resorbed nutrients are directly available for plant growth. It is postulated that in habitats with low nutrient availability, selection occurs for plant traits that result in higher nutrient conservation, including longer leaf and root life-span, higher sclerophylly and more efficient nutrient resorption from senescing organs [55, 56]. High level of organic matter supplied enough carbon, nitrogen and energy source to microbial growth. A close relationship has also been reported between soil fertility and microbial biomass [57, 58]. Internodes length of erect shoots, leaf width of erect shoots and reproductive branch height determined during the study have highly significant correlation (positive) with the soil available potassium. Exchangeable Mg and AN all were positively correlated with some morphological characters. Potassium (K), the third essential macro nutrient for higher plants, is involved in many important physiological processes in plants, and its functions have been shown to improve crop quality and the ability to resist adversities [59–61]. Plants from cold environments had significantly higher foliage N, P and Mg concentrations when grown in high latitudes, and that this may be an adaptive feature that enhances metabolic activity and growth rates under low temperatures of their native habitats [62, 63].

### The evolution trend of bermudagrass morphological characteristics at different latitudes

According to the results of analysis, a significant relationship was observed between the latitude and a number of bermudagrass morphological characteristics, indicating that latitude was the key factor influencing the evolution of *Cynodon*. Soil and climate factors play notable roles in the shaping of *Cynodon* phenotype as two important factors related to latitude. Phenotypic plasticity could result from heritable epigenetic effects that influence gene expression at different developmental stages or in different environments. Populations may need to respond to environmental change through phenotypic plasticity or adaptive evolution, by moving to a new area corresponding to environmental conditions they are adapted to, by genetically adapting to the new conditions, or by combinations of these responses. The complex evolutionary and breeding history has undoubtedly created both population structure and complex familial relationships [64]. Characterizing its germplasms for morphological characteristics diversity is an essential step in selection and breeding of this grass. These morphological characteristics such as leaf length, leaf width, turf height and reproductive branch height play important roles in the application of turfgrass. Bermudagrass in low- and high-latitude areas posses longer blade, higher turf height and reproductive branch height, which can be used to protect slope on both sides of highway and prevent soil erosion. *Cynodon* of mid-latitudes with lower turf height and reproductive branch height is excellent germplasm resources for lawn in the stadium and park. Thus, there are rich bermudagrass germplasm resources in different environmental conditions. Selecting representative germplasm resources in a wide range of geographical location can screen out more excellent germplasm resources. Due to long-term growth of wild bermudagrass in the bad environment, it goes through many generations of natural selection, and keep down features of survival and reproduction, which provide a rich material for breeding and breed improvement. The grass family is one of the most diverse plant taxa, and turf grasses have long been subjected to systematic studies using morphological characters [65]. Information resulting from this study, therefore, could be applied to various studies on bermudagrass such as genomics, cytogenetics, genetics and breeding.

## Conclusions

In this study, these analyses against *C. dactylon* morphological traits and environment factors have revealed a remarkable variety of phenotypic resources at different latitudes which is important for the reserve and propagation of native *Cynodon* species. We also found that larger *Cynodon* morphological sizes were strongly associated with low- and high-latitudes among the 310 *Cynodon* accessions. For adaptive phenotypic plasticity to evolve, individuals must be capable of responding to latitude, and plastic genotypes must have a fitness advantage over genotypes that are incapable of altering their phenotypes. Our findings expand the current knowledge on morphological diversity of turf-type wild bermudagrass. Accessions of *C. dactylon* at different latitudes may further enrich the gene pool and adapt to more different environments, which are influenced by latitude-related soil characters such as soil available potassium, exchangeable Mg and available nitrogen and climate factors such as annual average precipitation and annual average temperature. The variation present in the *Cynodon* accessions may have significant contribution in *C. dactylon* breeding programs for various goals such as turf, forage, soil stabilization and remediation, as well as our understanding of the evolution of warm season grass.

## Abbreviations

AK: Available Kalium
AN: Available Nitrogen
ANOVA: One-way analysis of variance
CA: Cluster Analysis
CV: Coefficient of variation
P: Probability
PCA: Principal Component Analysis
PCs: Principal components
r: Correlation coefficients
SD: Standard deviation
SOM: Soil Organic Matter
TN: Total Nitrogen
UPGMA: Unweighted pair group mean average
